# Transient myeloproliferative disorder in a newborn with down syndrome treated with rasburicase for the risk of development of tumor lysis syndrome: A case report

**DOI:** 10.1186/1752-1947-5-407

**Published:** 2011-08-24

**Authors:** Athanasios Tragiannidis, Zoe Dorothea Pana, Theodotis Papageorgiou, Emmanuel Hatzipantelis, Maria Hatzistilianou, Fani Athanassiadou

**Affiliations:** 1 Second Pediatric Department, Aristotle University of Thessaloniki, Ahepa General Hospital, Thessaloniki, Greece

## Abstract

**Introduction:**

Transient myeloproliferative disorder is a hematologic abnormality characterized by an uncontrolled proliferation of myeloblasts in peripheral blood and bone marrow that primarily affects newborns and babies with Down syndrome. Tumor lysis syndrome is rarely associated with transient myeloproliferative disorder.

**Case presentation:**

Transient myeloproliferative disorder was diagnosed in a seven-day-old baby girl with Down syndrome, who was referred to our department due to hyperleukocytosis. Our patient developed tumor lysis syndrome, successfully treated with rasburicase, as a complication of transient myeloproliferative disorder resulting from rapid degradation of myeloid blasts after initiation of effective chemotherapy.

**Conclusions:**

Tumor lysis syndrome is rarely reported as a complication of transient myeloproliferative disorder. To the best of our knowledge, this is the first case of a newborn with Down syndrome and transient myeloproliferative disorder treated with rasburicase for developing tumor lysis syndrome.

## Introduction

Transient myeloproliferative disorder (TMD) of Down syndrome (DS), also known as transient abnormal myelopoiesis, characteristically manifests in the first few days of life with numerous circulating blast cells exceeding the number of blast cells in the bone marrow and with spontaneous or no resolution within a few weeks [1-3]. Occasionally, however, TMD has preceded acute megakaryoblastic leukemia (AML-M7) after a period of remission lasting several months to years [4-6]. Tumor lysis syndrome (TLS) is rarely reported after initiation of effective chemotherapy as a complication of transient myeloproliferative disorder [7].

In this report, we present a case of a baby with DS and TMD who developed TLS successfully treated with rasburicase.

## Case presentation

A seven-day-old Caucasian baby girl with DS was referred to our department due to hyperleukocytosis. The baby was born by vaginal delivery at 39 weeks and two days of gestation. Her Apgar scores were 8 and 9 at one and five minutes, respectively. Her birth weight and body length were 3420 g and 49 cm, respectively. Our patient showed typical signs consistent with trisomy 21 confirmed by karyotype analysis (47, XX, +21). The second day after birth, she presented to our facility with reduced feeding intake. Peripheral blood test results revealed a white blood cell (WBC) count of 70.8 × 10^9 ^cells/mm^3 ^with 70% of blasts. Due to hyperleukocytosis our patient was referred to the Hematology Oncology Unit of the Second Pediatric Department of Aristotle University of Thessaloniki. On admission our patient had hepatosplenomegaly, abdominal distension, hypotonia and dyspnea, probably due to hyperviscosity. Echocardiography revealed a ventricular septal defect (VSD), pericardial effusion and persistent pulmonary hypertension of newborn (PPHN). An ultrasound scan of the abdomen confirmed hepatosplenomegaly with an increased echo signaling of the liver. Biochemical data revealed increasing levels of lactate dehydrogenase (LDH) (2027IU/L), serum inorganic phosphorus (P) (6.0 mg/dL) and uric acid (UA) (4.2 mg/dL). Results of blood gas analysis performed on admission were in the normal ranges. The laboratory hematological, biochemistry and coagulation test results on admission are shown in Table 1. Bone marrow aspiration revealed hypercellular marrow with an excess of blasts. Antigenitically, the blasts were negative for myeloperoxidase, positive for CD33, CD34 and CD61, and showed weak coexpression of CD7. The lymphoid and erythroid populations exhibited no antigenic aberrations or atypicality. On the basis of the blast cell immunophenotype, the presentation suggested a diagnosis of TMD. Our patient was put on antileukemic treatment with cytosine-arabinoside (Ara-C) (1.5 mg/kg per day for eight consecutive days) (day three) due to persistence of hyperleukocytosis and deterioration of respiratory function (dyspnea, tachypnea). Parental written informed consent was obtained prior to chemotherapy initiation. Biochemistry test results after initiation of antineoplastic treatment (day four) revealed increased levels of serum potassium, serum inorganic phosphorus and a marginally elevated level of uric acid (Table 1). Due to the fact that our patient had hyperleukocytosis and elevated LDH levels that meant a high tumor burden, we decided to treat her for the increased risk of TLS development. Therefore, she was put on rasburicase (0.2 mg/kg), fluid therapy and forced diuresis treatment immediately. Metabolic parameters were normalized seven days after the initiation of rasburicase treatment (day 10). Laboratory hematological, biochemistry and coagulation test values on day four (initiation of rasburicase) and 10 (end of rasburicase administration) are shown in Table 1. Peripheral blasts disappeared by day eight after the initiation of Ara-C. Due to hepatosplenomegaly and pericardial effusion, she developed mild respiratory distress without requiring ventilation. Our patient was discharged at the age of six weeks with no further complications and is currently well at the age of nine months.

**Table 1:** Laboratory hematological, biochemistry and coagulation test values from our patient on admission, on day four (initiation of rasburicase) and day 10 (end of rasburicase administration)

	Day 1	Day 4	Day 10
White blood cell count (cells/μL)	70,800	43,500	4970
Hemoglobin (g/dL)	17.1	15.8	15.0
Hematocrit (%)	48.8	46.0	42.8
Platelets (cells/μL)	248,000	214,000	118,000
Total protein (g/dL)	4.90	4.74	5.16
Albumin (g/dL)	3.25	3.26	3.18
Lactate dehydrogenase (IU/L)	2027	2235	681
Urea (mg/dL)	20	18	15
Creatinine (mg/dL)	0.27	0.41	0.24
Sodium (mEq/L)	140	140	137
Potassium (mEq/L)	5.8	5.4	4.5
Phosphorus (mg/dL)	6.0	5.2	2.8
Calcium (mg/dL)	9.07	8.61	10.51
Uric acid (mg/dL)	4.2	4.0	0.8
Alanine aminotransferase (IU/L)	37	34	29
Aspartate aminotransferase (IU/L)	14	20	20
Prothrombin time (seconds)	13.8	12.0	12.2
Activated partial thromboplastin time (seconds)	32.2	28.90	30.5
Fibrinogen (mg/dL)	279	253	266

## Discussion

We describe the case of a newborn with DS and TMD who developed TLS that was successfully treated with rasburicase. To the best of our knowledge this is the first case of a newborn with DS and TMD treated with rasburicase for preventing the occurrence of TLS. Usually, transient leukocytosis associated with DS is generally diagnosed in the first few weeks of life. TMD, also known as transient leukemia, occurs in about 10% of neonates with DS [6]. It is often accompanied by hepatosplenomegaly, pericardial and pleural effusions, hepatic disease, as in our patient and a pustular rash [6]. Although TMD resolves in the majority of DS babies, 20% to 30% subsequently go on to develop AML-M7, usually within in the first 4 years of life [4,6]. AML develops either by overt progression or after an apparent remission of TMD with AML arising many months later, presumably from a subcolony of persisting TMD cells that acquire a selective advantage.

Most neonates with TMD do not need chemotherapy as the clinical and laboratory abnormalities spontaneously resolve within three to six months after birth. However, symptomatic babies with TMD, especially those with high blast counts or liver dysfunction, may benefit from low-dose cytosine arabinoside. Chemotherapy is usually given at the treating physician's discretion and various groups have reported similar dosage schedules and response. In the Pediatric Oncology Group (POG) study 9481, 10 mg/m^2 ^per dose or 1.2 to 1.5 mg/kg per dose was given subcutaneously or intravenously by slow injection twice a day for seven days [8]. In the AML-BFM study, 0.5 to 1.5 mg/kg was administered for 3 to 12 days [9]. As TMD blasts are highly sensitive to cytarabine, there is generally a rapid response, characterized by the disappearance of peripheral blasts by day seven of treatment. However this is not always the case, especially in babies with severe liver disease associated with fibrosis. Here, the response to chemotherapy is poor and overall there is a poor prognosis. Overall, TMD has been reported to have a mortality rate of approximately 20% [8,9].

TLS is a group of metabolic complications that can occur after treatment for cancer, usually lymphomas and leukemias, and sometimes even without treatment. These metabolic complications include hyperkalemia, hyperphosphatemia, hyperuricemia and hyperuricosuria, hypocalcemia, and consequent acute uric acid nephropathy and acute renal failure [7]. There are only few reports of TLS associated with TMD [10,11]. Abe *et al*. reported the case of a neonate with DS who developed acute renal failure secondary to hypotension and TLS as a complication of TMD. The patient was treated with diuretics and pressor agents, but unfortunately died [11]. Kato *et al*. reported the case of a baby with DS who developed TLS as a result of TMD, successfully treated with allopurinol and diuretics [10].

## Conclusions

The clinical course of our patient indicates that TLS may develop in cases with DS and TMD. Intensive supportive and prophylactic therapy for preventing TLS should be given in cases of TMD showing prominent circulating blasts. To the best of our knowledge this is the first case of a newborn with DS and TMD treated with rasburicase for developing TLS.

## Consent

Written informed consent was obtained from the patient's next-of-kin for publication of this case report and any accompanying images. A copy of the written consent is available for review by the Editor-in-Chief of this journal.

## Competing interests

The authors declare that they have no competing interests.

## Authors' contributions

AT made the diagnosis of hematological disease and wrote the case report. ZDP contributed to the writing and the diagnostic procedures of the case report. TP analyzed and interpreted the data from our patient regarding the hematological disease and was responsible for our patient's treatment and care. EH was responsible for our patient's treatment and follow-up. MH analyzed and interpreted the data from our patient regarding the immunophenotype of the hematological disease. FA analyzed and interpreted the data from our patient regarding hematological disease, and was responsible for our patient's treatment and care as well as for the writing and revision of the manuscript. All authors contributed equally to the final draft of the manuscript, and read and approved the final manuscript.
